# The Effects of Diagnostic Anatomical Features on Treatment and Recovery in Feline Spinal Trauma

**DOI:** 10.3390/vetsci13090949

**Published:** 2026-09-12

**Authors:** Mehmet Kılınç, Sadık Yayla

**Affiliations:** 1 Anatomy Department, Veterinary Faculty, Dicle University, 21280 Diyarbakır, Türkiye; 2 Surgery Department, Veterinary Faculty, Dicle University, 21280 Diyarbakır, Türkiye; sadik.yayla@dicle.edu.tr

**Keywords:** cat, diagnostic anatomy, polyaxial screw, SOP plate, spinal trauma, vertebral instability

## Abstract

**Background:** Spinal trauma remains a clinically significant condition in small animal practice. A thorough understanding of spinal anatomy is essential for accurate lesion localization, appropriate implant selection, optimal surgical planning, and improved treatment outcomes in feline spinal trauma. **Aim:** The aim of this study is to evaluate treatment strategies and outcomes in cats with spinal trauma by integrating diagnostic anatomical features with surgical planning. **Methods:** A total of 20 cats were included, and the location, type, and severity of the spinal lesions were determined through comprehensive clinical, radiographic, neurological, and orthopedic examinations. Surgical stabilization was performed using a polyaxial screw–rod system for thoracic vertebral injuries and a String-of-Pearls (SOP) plate for lumbar vertebral lesions. The type and size of implant used during these procedures were determined according to the anatomical characteristics in each case. In cases with spinal cord compression, decompressive procedures, including laminectomy or hemilaminectomy, were carried out as indicated. **Results:** The results demonstrated that the surgical stabilization and decompression techniques used in this study provided satisfactory spinal stability and significant functional improvement in most cats. **Conclusions:** This is related to a good understanding of the lesion type and the anatomical features of the affected spine, as these anatomical features determine not only surgical planning but also the method and implant size to be used.

## Simple Summary

Spinal trauma is a clinically significant condition in small animal practice. Therefore, knowledge of spinal anatomy contributes to appropriate implant selection, optimal surgical planning, and improved treatment outcomes during spinal surgery. This study evaluated treatment strategies and outcomes in cats with spinal trauma by integrating diagnostic anatomical features with surgical planning. In this study, which included a total of 20 cats with spinal trauma, surgical treatment was planned after clinical, radiographic, neurological, and orthopedic examinations. The type and size of the implant used during these procedures were determined according to the anatomical characteristics in each case. This approach has been shown to provide a satisfactory level of spinal stability and significant functional improvement in spinal trauma cases. This is possible with a good understanding of the anatomical features of the affected spine and the type of lesion.

## 1. Introduction

Spinal trauma remains a highly relevant topic in small animal practice. Successful management of these cases requires not only accurate diagnosis and appropriate therapeutic decision-making but also a thorough understanding of spinal anatomy by surgeons involved in neurosurgery. Comprehensive anatomical knowledge facilitates precise lesion localization, appropriate implant selection, and meticulous surgical planning. Consequently, integrating diagnostic anatomical evaluation into the clinical assessment may contribute to improved treatment strategies and increase the likelihood of favorable surgical and neurological outcomes in cats with spinal trauma [1,2,3,4].

In general, vertebral fractures and dislocations account for approximately 10% of all neurological cases in cats [5,6].

The rapid and effective application of neurological examination procedures in spinal trauma patients is an important aspect of the successful management of these cases [7,8,9]. In order to prevent or minimize secondary spinal cord injury following trauma, the respiratory and circulatory functions of the patients should be stabilized [10].

Lumbar vertebrae are most commonly affected by spinal trauma, followed by sacrococcygeal, thoracic, and cervical vertebrae. In addition, there is a significant incidence of fractures or dislocations occurring at the thoracolumbar and lumbosacral junctions [11,12,13].

In vertebral fractures, compression of the spinal cord occurs due to hematoma, traumatic disc extrusion or dislocated bone fragments, or compromised stability. Eliminating this compression and achieving proper stabilization is the main goal of surgical treatment of vertebral fractures and dislocations [1,8,10,12,14].

Vertebral fractures and dislocations can be treated both surgically and conservatively [12,15]. Conservative treatment includes splinting, bandaging, external stabilization, cage rest, exercise restriction, and steroid administration [11,16].

Many different stabilization techniques have been described to stabilize the vertebral column. Among these, stabilization of the spinous processes by plate application, stabilization of the vertebral bodies with plates, stabilization with polymethyl methacrylate (PMMA) with pin or screw application, stabilization using external fixators, pin-cerclage applications, and stabilization using pedicle screws and rod systems are commonly used [12,13]. Successful implementation of these procedures requires a thorough understanding of spinal anatomy and considerable surgical experience.

The aim of this study is to evaluate treatment strategies and outcomes in cats with spinal trauma by integrating diagnostic anatomical features with surgical planning.

## 2. Materials and Methods

### 2.1. Animal Material

This study was conducted at the Surgery Clinic of the Animal Hospital, Faculty of Veterinary Medicine, Dicle University, between 2022 and 2025. A total of 20 cats of various breeds, ages, and sexes diagnosed with spinal trauma were included. The cases were comprehensively evaluated with respect to the etiology of the injury, the localization of spinal lesions, the treatment modalities applied, and clinical outcomes.

### 2.2. Clinical Examination

Cases were selected based on the history, clinical presentation, neurological examination, and radiographic findings. Cats presenting with neurological deficits consistent with spinal trauma, including paresis, paraplegia, or sensory impairment, were included in the study. For each cat, breed, age, sex, body weight, cause of injury, concomitant injuries, and the interval between hospital admission and surgery were recorded. A complete physical and orthopedic examination was performed to identify spinal injury and any concurrent non-neurological lesions. A comprehensive neurological examination was conducted in all cats, including assessment of spinal reflexes, spinal palpation for pain or hyperesthesia, superficial and deep pain perception, urinary and fecal continence, and the presence of muscle atrophy. Deep pain perception was assessed by applying firm pressure to the digits and interdigital skin using forceps. Deep pain perception was considered present when a conscious behavioral response, such as vocalization, turning of the head, or an attempt to bite, was observed. Cats showing no response or only withdrawal reflexes without conscious recognition were considered negative for deep pain perception.

Each cat was neurologically graded according to the classification system described by Grasmueck and Steffen (2004) [17]. The neurological grades were defined as follows:

Grade I: Spinal pain without neurological deficits.
Grade II: Ambulatory paraparesis with normal urinary function.
Grade III: Ambulatory paraparesis with urinary retention.
Grade IV: Non-ambulatory paraparesis or paraplegia with urinary retention and preserved deep pain perception.
Grade V: Paraplegia with urinary retention and absence of deep pain perception.

### 2.3. Radiography

Standard latero-lateral and ventro-dorsal radiographs were taken during the radiographic examination.

### 2.4. Anesthesia, Analgesia, and Antibiotic Therapy

Each case was induced with intubation following injection of medetomidine (Domitor^®^, Orion Pharma, Espoo, Finland) at a dose of 80 μg/kg IM, butorphanol (Butomidor^®^ 10 mg/mL, Richter-Pharma, Wels, Austria) at a dose of 0.4 mg/kg SC, and ketamine (Ketasol^®^, Richter-Pharma, Austria) at a dose of 5 mg/kg IM, and anesthesia was maintained with isoflurane and 100% oxygen (as previously reported [18,19]).

To provide multimodal analgesia, 12 mg ketamine, 4.8 mg butorphanol and 40 mcg medetomidine, as previously described by different authors [19,20], were diluted in 100 mL of 0.9% isotonic sodium chloride solution and administered intravenously by infusion using an automatic infusion pump at a rate of 5 mL/kg per hour before, during and after surgery.

All patients were started on antibiotic prophylaxis with intravenous (IV) cefazolin sodium (Cezol, Deva, Şahinbey, Turkey) at a dose of 20–22 mg/kg before the start of surgery and continued for 5 days postoperatively [19,20].

### 2.5. Implants and Instruments

In all cases, vertebral stabilization was performed using either a polyaxial screw–rod system (Hasvet Medikal A.Ş., Antalya, Türkiye) or a 2.0 or 2.7 mm SOP plate system (Orthocat Vet, Istanbul, Türkiye). When indicated, spinal cord decompression was achieved by dorsal laminectomy or hemilaminectomy using a high-speed surgical drill fitted with diamond burs.

### 2.6. Surgical Procedure

**Figure 1:**
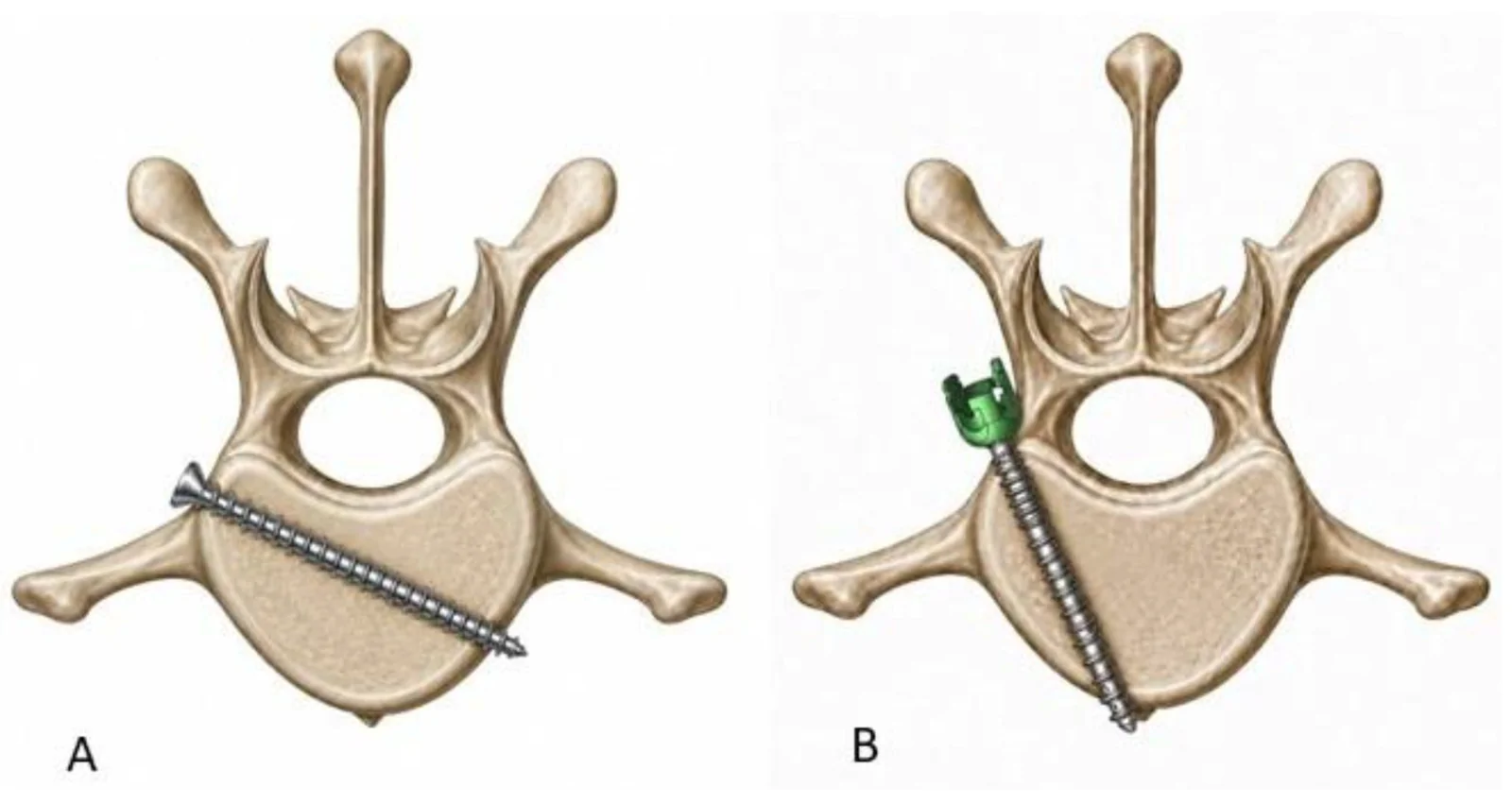
Screw locations on the vertebrae: (**A**) screw for SOP plate and (**B**) polyaxial screw (AI was used in the drawing of this figure).

The animals were placed in the sternal position, and their legs were freely extended on the table. After the area was demarcated with a sterile drape, a skin incision was made along the midline of the back, extending from the lesion site to at least two cranial and two caudal vertebrae. The skin incision and dissection of the subcutaneous fascia were performed, taking care to preserve the attachments of the m. erector spinae to the vertebral processes. After making the dorsal fascial incision, the unstable vertebrae were reduced using double-ended bone forceps, and stabilization was initiated. These forceps were placed on the spinous processes or vertebral body, and reduction was achieved by gently applying upward traction to ensure normal anatomical alignment of the spine. To stabilize instable vertebrae, stabilization was achieved by placing at least two screws cranial and caudal to the affected vertebra. Due to their anatomical structure, a polyaxial screw–rod system was used for thoracic vertebrae, while an SOP plate was preferred for lumbar vertebrae. The screw holes were positioned dorsolaterally to ventromedially, above the points where the transverse processes join the vertebral bodies (Figure 1A,B). During these procedures, the anatomical structure and characteristics of the affected vertebra were taken into account, which was particularly influential in determining the diameter and length of the screws.

### 2.7. Postoperative Evaluation

During postoperative checks, bilateral radiographs were taken laterolaterally or ventrodorsally to assess implant position and fracture reduction. The results obtained during the postoperative evaluation phase were categorized as poor outcome, functional recovery, and good recovery [12,19]. Following surgery, the cats were typically hospitalized for approximately 2 days. Hospitalization was extended when additional clinical monitoring or supportive care was considered necessary. During hospitalization, general clinical and neurological status, voluntary motor function, urinary function, appetite, and the surgical site were regularly monitored. Following discharge, activity restriction and cage rest were recommended, and the cats were reassessed through clinical, neurological, and radiographic follow-up examinations. Poor outcome was defined as persistent difficulty in urination and/or no improvement in gait after treatment. Functional outcome was considered to be when the cat could urinate and regained the ability to walk. Good outcome was defined as normal urination, gait, and the absence of proprioceptive abnormalities.

## 3. Results

According to the data in our study, all cats were found to have suffered spinal trauma due to high-rise syndrome, such as falling from a balcony or window.

All cases (20 cats) showed varying degrees of neurological dysfunction, with grade III in three cats, grade IV in nine cats, and grade V in eight cats (Table 1). Urinary retention was observed in all cases (100%). In cats with grade III neurological dysfunction, functional improvement was observed after treatment. Of the cats classified as grade IV neurological dysfunction, seven showed functional improvement, and two showed excellent improvement. Of the cats classified as grade V neurological dysfunction, four showed poor improvement, one showed functional improvement, and it was noteworthy that three cats died before follow-up could be conducted. Apart from these three deaths, no additional major intraoperative or postoperative complications were recorded. Specifically, no surgical-site infection, wound dehiscence, clinically evident implant loosening or failure, or requirement for revision surgery was observed during the follow-up period.

**Figure 2:**
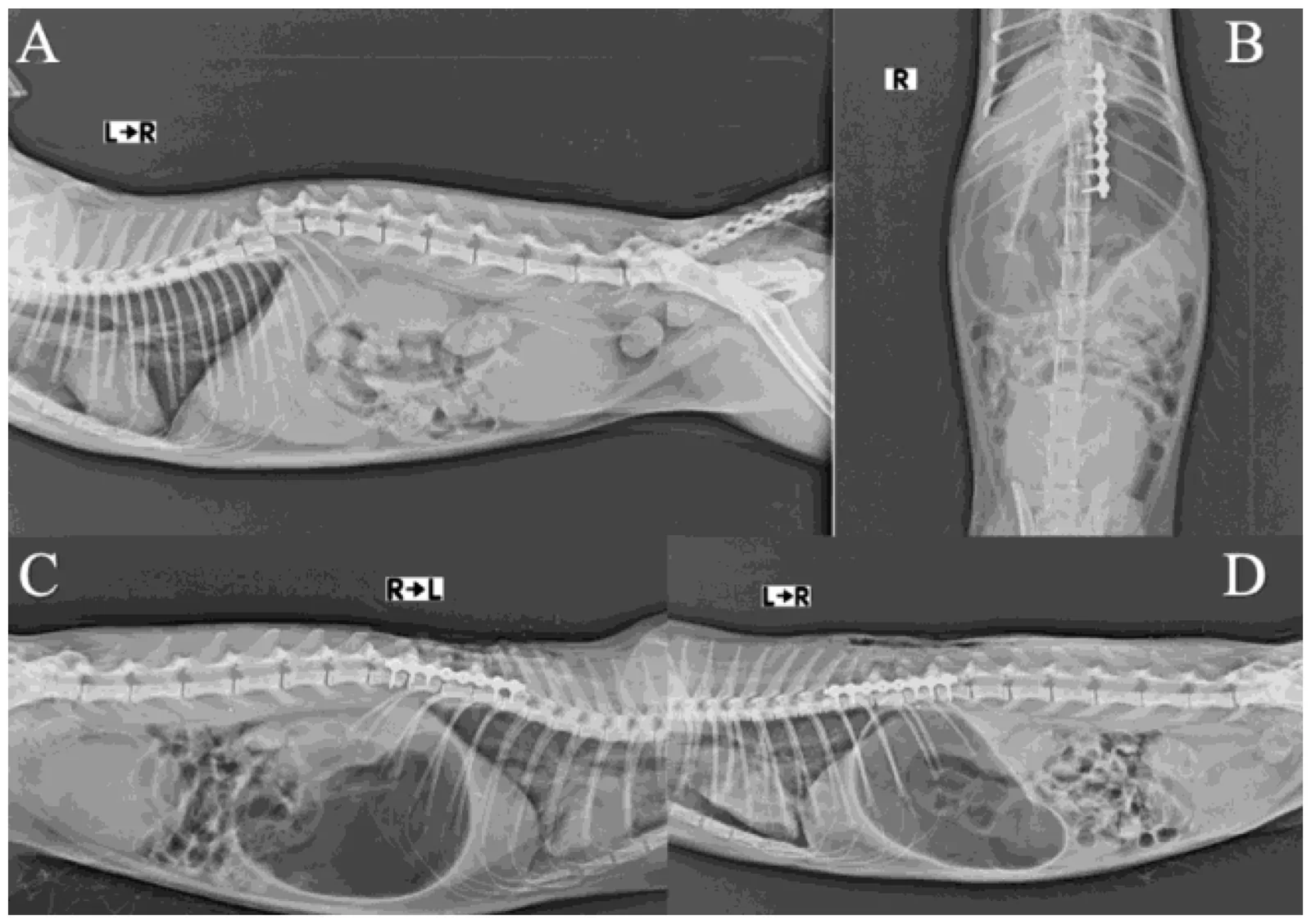
Radiographs of case number fourteen: preoperative left lateral (**A**), postoperative ventrodorsal (**B**), right lateral (**C**) and left lateral (**D**) radiographs.

**Figure 3:**
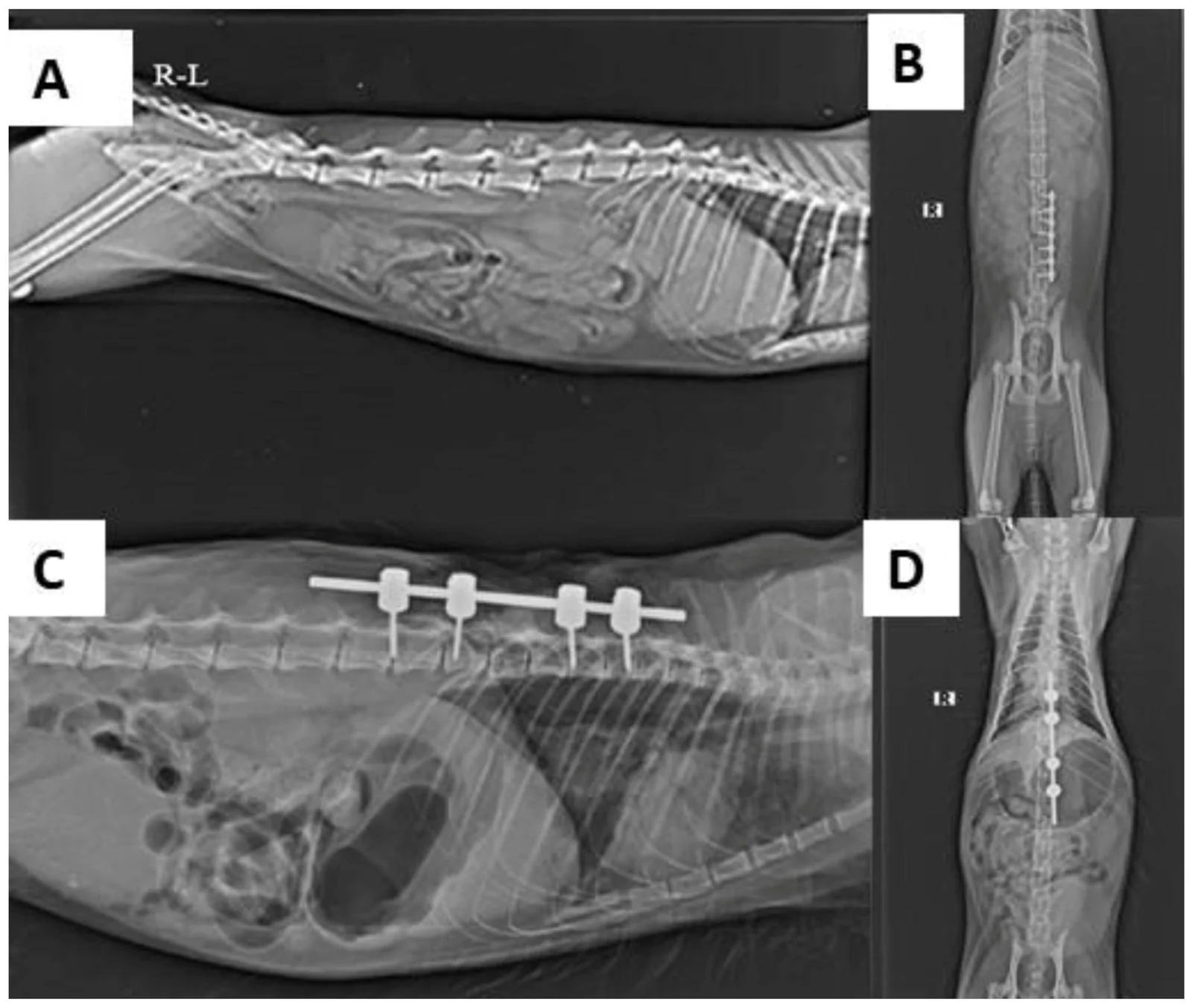
Radiographic images from various cases. (**A**) L2 caudal end plate fracture, (**B**) stabilization with SOP plate in a different case, (**C**) fixation with polyaxial screw–rod system in another case (L/L view), and (**D**) fixation with polyaxial screw–rod system (V/D view).

**Figure 4:**
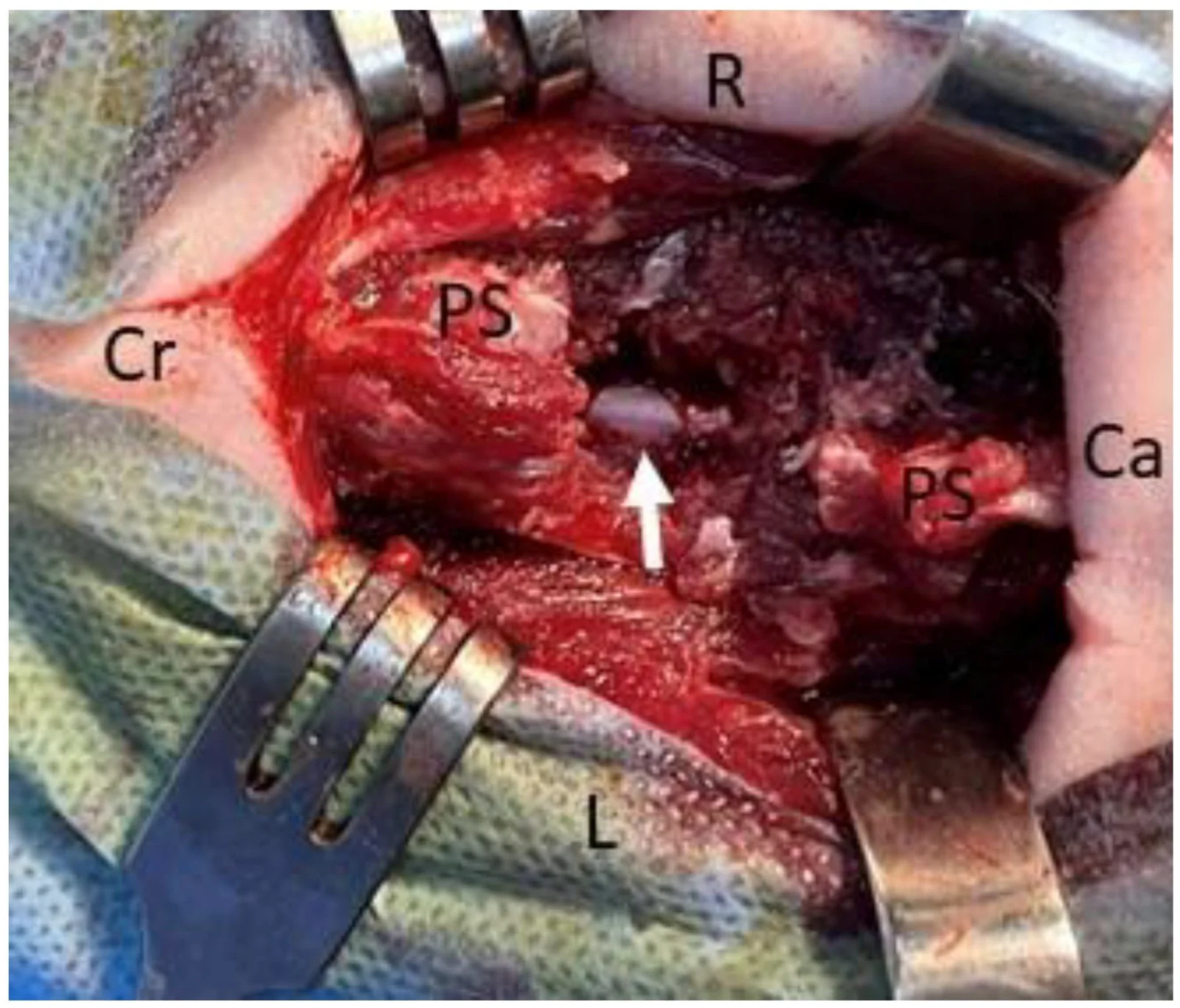
Appearance of the spinal cord after dorsal laminectomy during surgery (Cr: cranial, Ca: caudal, R: right, L: left, PS: spinose process, white arrow: spinal cord).

The median age of the cats was 2.5 years (range, 5 months to 4 years). Of the nine male cats, seven (77.8%) were neutered and two (22.2%) were sexually intact, whereas eight (72.7%) of the 11 female cats were spayed and three (27.3%) were sexually intact. In all cases (20 cats), there was instability and compression on the spinal cord due to vertebral body or end plate fractures or dislocations. Therefore, the primary goal was spinal cord and nerve root decompression and vertebral stabilization. For this purpose, polyaxial screws were applied in 11 cases and SOP plates in nine cases (Figure 2 and Figure 3). For vertebral decompression, dorsal laminectomy was performed in two cases (Figure 4) and hemilaminectomy in one case (Table 2).

In this study, three out of a total of 20 cats with vertebral instability (15%) had end plate fractures. The distribution of vertebrae with end plate fractures was as follows: one cat had L4 cranial, one cat had L2 caudal, and one cat had L6 caudal. In other cases, vertebral fractures or dislocations were not observed in the C1-C5 and C6-T2 regions, while they were found in the T3-L3 region in 13 cases (65%) and in the L4-S1 region in seven cases (35%). No lesions were found in the sacrococcygeal region (Table 2). Concurrent fractures of the pelvis and appendicular skeleton were identified in four cats (20%), including one scapular fracture, two pelvic fractures, and one radius–ulna fracture. Given the potential for progressive neurological injury associated with vertebral instability and spinal cord compression, neural decompression and stabilization of the affected vertebral segment were considered the primary surgical priorities. Following completion of the spinal procedure, all concurrent fractures were surgically treated during the same anesthetic session, thereby avoiding an additional anesthetic episode and enabling comprehensive management of the traumatic injuries.

## 4. Discussion and Conclusions

According to the data in this study, in terms of trauma, all cases (100%) of the cats were due to high-rise syndrome, specifically falls from balconies or windows. This once again confirms that spinal injuries in cats are highly prevalent (84%) due to high-rise syndrome, as reported in other studies [12,13,21], and therefore, cat owners should be informed and reminded about taking the necessary precautions. High-rise syndrome was not expected to be the cause of trauma in all cases observed in this research, but the presence of vertical structures in cities may help explain this high incidence.

In cases of spinal trauma, paraplegic patients with conditions such as degenerative discs, cord injury, or spontaneous herniation have been reported to have a good prognosis if timely decompression is performed [12,17,22]. Therefore, appropriate transfer of the patient to a post-traumatic center, followed by trauma management and damage repair, is of paramount importance. In our study, it was observed that the time to hospital admission for all cases was more than 24 h and varied between 24 and 72 h. It is recommended that this period be brought forward as much as possible.

**Figure 5:**
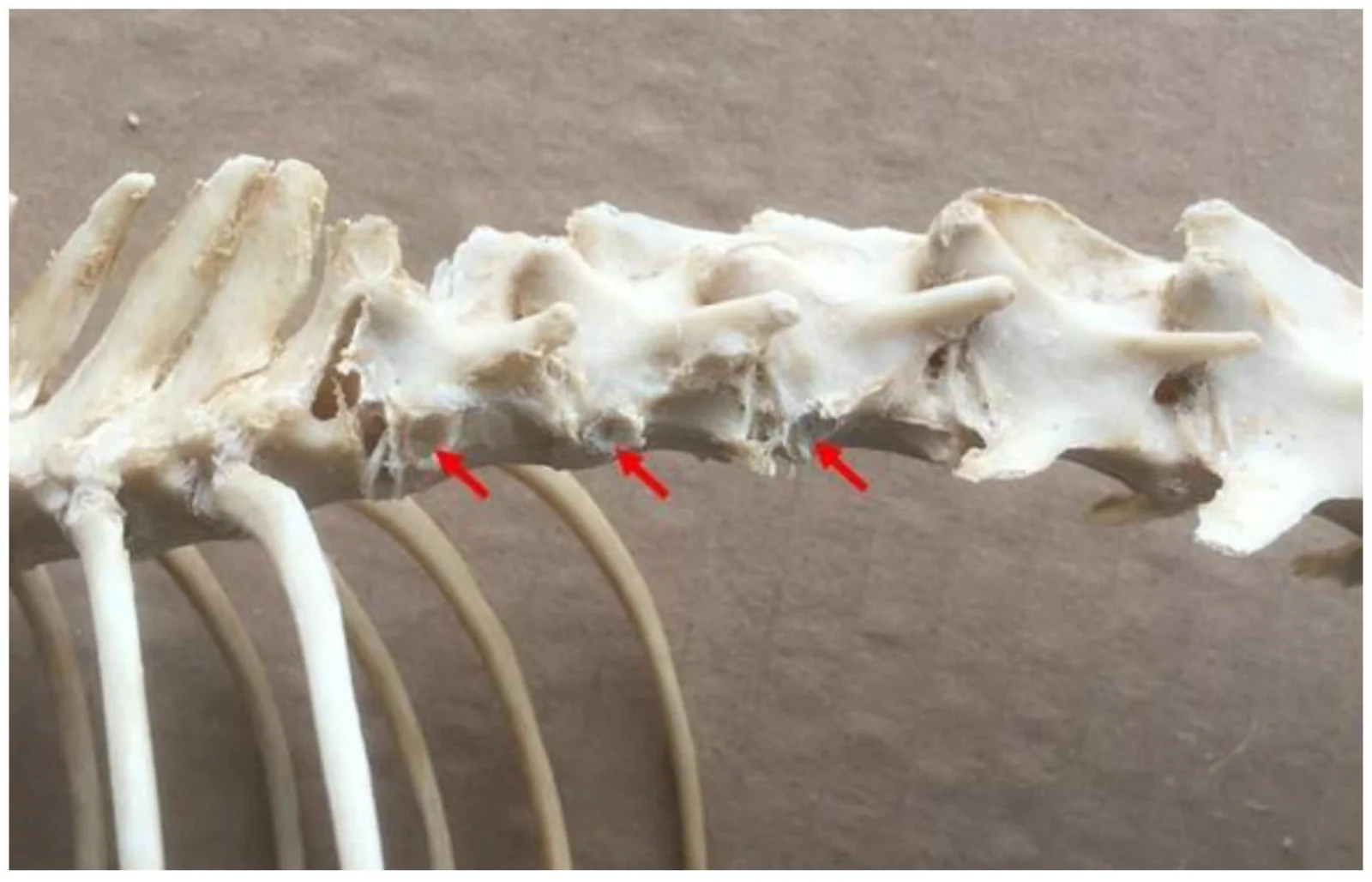
Thoracolumbar junction of the spine: view of the costal fovea in the anterior part of the body of the last three vertebrae (arrows) (from the first author’s (MK) own archive).

The most frequently affected region in spinal injuries is the thoracolumbar junction T11-T13 and L1, followed by the last lumbar vertebra and the lumbosacral junction. Yayla et al., 2023, reported [12] that the most frequently affected region in cats with spinal fractures is the L4-L7 region, accounting for 48%. This is followed by the T3-L3 region, which accounts for 46% of spinal fractures in cats. The C6-T2 region constitutes 3% of spinal fractures in cats, while the S1-S3 region has a rate of 1%. In our study, no cases with damage in the C1-C5 and C6-T2 regions were found, while 13 cases (65%) had damage in the T3-L3 region, and seven cases (35%) had damage in the L4-S1 region. The reason why the thoracolumbar region is most frequently affected by trauma can be attributed to the fact that this segment is anatomically less flexible and unprotected. Injuries in this region usually result in paraplegia or paraparesis. Cervical injuries in cats are rare but can cause more-serious neurological loss [23]. In the thoracic vertebrae, on the cranial and caudal edges of each side, there are cranial and caudal costal foveas for the articulation of the ribs. The caudal fovea of the cranial vertebra and the cranial fovea of the caudal vertebra meet, joining with the edge of the intervertebral disc, thus forming an articular fossa. This fossa articulates with the costal cap (art. capitis costa). The intraarticular ligament (lig. capitis costa intraarticulare and lig. intercapitale) connecting the costal cap located here to the costal cap on the opposite side contributes to spinal stability. The lateral aspect of the anterior portion of the body of the last three vertebrae contains a costal fovea, with which the costal cap articulates. The reason why the thoracolumbar region is most frequently affected is thought to be the anatomical presence of one costal fossa on the lateral side of the cranial part of the vertebral body, especially the last three vertebrae, and the articulation of the costal head only with this fossa, the absence of the last two ribs in the costal arch, and their location as asternal ribs within the abdominal muscles. One of the reasons for the high frequency of TL spinal trauma is that this is the middle of the spine, and it experiences the greatest bending moment (Figure 5).

It has been reported [3,11] that various techniques used for vertebral instability include the use of polyaxial screws, stabilization with polymethyl methacrylate (PMMA), and plate applications [14,15]. This study includes the use of polyaxial screws and plate applications for stabilization. Polyaxial screws were preferred for the thoracolumbar region due to kyphosis in cats, while SOP plate application was preferred for the lumbar region due to the more even arrangement of the vertebrae.

It is known that urinary problems such as retention or incontinence are common in patients who have suffered spinal trauma [4,8]. The prognosis tends to be worse in patients with bladder involvement. Most patients with grade V neurological damage are recommended for euthanasia 24–48 h after losing deep pain perception and poor prognosis [16,22]. Furthermore, it has been reported that 6–15% of patients with vertebral column injury also have concomitant abdominal injury [16]. This can result in hemoperitoneum, uroperitoneum, or gastrointestinal or hepatobiliary injuries. In our study, abdominal trauma resulted in abdominal hernia in two cases and mild liver hematoma in another. Simultaneous fractures of the pelvis and appendicular skeleton have been reported in 14–48% of animals with vertebral fractures or dislocations [12,16]. In our study, three cases involved thoracic trauma, one patient had a scapular fracture, one patient had an antebrachium (radius + ulna) fracture, and two patients had pelvic fractures. Simultaneous injuries (such as pneumothorax, appendicular fractures, or hemoperitoneum) have been reported to be more common in cats (83% in cats, 66% in dogs) [22]. In our study, simultaneous fractures of the pelvis and appendicular skeleton were detected in 20% of animals with vertebral fractures or dislocations.

Grasmueck and Steffen (2004) reported [17] that, in surviving cats, clinical outcomes were complete (grade II to IV) in 12 cases, functional (grade III to IV) in three, and poor (grade III to IV) in three. They reported no clear correlation between the degree of initial neurological dysfunction and outcome in this relatively small population. However, if deep pain perception is unaffected in cats with spinal cord injury, a favorable prognosis can be concluded. In our study, recovery was functional in cats with grade III neurological dysfunction (three cats). Of the cats classified as grade IV neurological dysfunction (nine cats), seven recovered functionally, and two recovered completely. Of the eight cats with grade V neurological dysfunction, four showed poor recovery, one showed functional improvement, and three died within a week of treatment.

Ensuring adequate blood flow and maintaining oxygenation is crucial for systemic stabilization of the spinal cord. Therefore, intravenous fluid therapy and oxygen supplementation are necessary to prevent secondary spinal cord injury following a traumatic event (primary injury) [3,10]. Depending on the level of spinal column damage, secondary spinal cord injury is reported to result from loss of local or systemic blood pressure regulation and decreased spinal cord blood flow caused by vascular damage [3,24]. In our study, adequate blood circulation and oxygenation for systemic stabilization were attempted through intravenous fluid therapy and oxygen supplementation.

Three cases died within one week of treatment. Because the study involved a relatively small population, a clear correlation between the degree of neurological dysfunction and the outcome could not be established. However, it can be said that cats with spinal cord injury have a favorable prognosis if their deep pain perception is intact.

**Figure 6:**
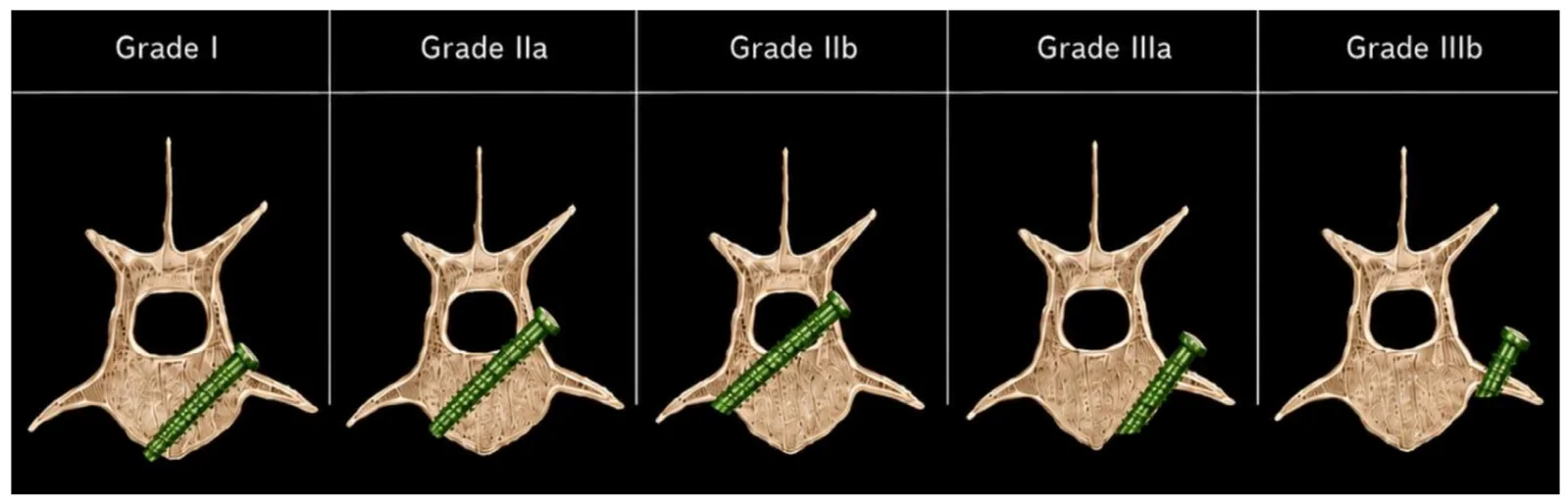
Modified Zdichavsky classification with grade I corresponding to a non-breached drill tract, grade IIa denoting partial breach of medial pedicle wall, grade IIb corresponding to full breach of medial pedicle wall, and grades IIIa and IIIb denoting partial and full breach of lateral pedicle vertebral body (from Mullins et al., 2024 [25]), (AI was used in the drawing of this figure).

Spinal surgery can be challenging for most practitioners, especially as it requires experience in addition to a good surgical approach and anatomical knowledge [12]. The Zdichavsky classification, modified by Mullins et al. (2024) [25], identifies and figurate correct and incorrect practices during spinal surgery (Figure 6). Permanent neurological damage is inevitable during these incorrect practices. Furthermore, because the screws are inserted from the right to the left side of the vertebra, where the descending aorta lies in close proximity, careful attention should be paid to the drill bit and screw lengths during preparation of the screw tract to avoid vascular injury. Therefore, unless there is a contraindication, we recommend that the approach for the SOP plate or polyaxial screw–rod system be performed from the left side of the spine.

The main limitations of this study were the relatively small number of cases and the inclusion of only cats that had sustained spinal trauma following high-rise syndrome. Therefore, the findings may not be directly generalizable to spinal injuries caused by other trauma mechanisms. In addition, differences in lesion location, neurological severity, and the stabilization system used limited subgroup comparisons. Larger multicenter studies including different trauma mechanisms are required to confirm these findings. There was also a lack of CT imaging, as a CT scanner was not available at the veterinary hospital where the study was conducted. CT imaging could have enabled a more detailed characterization and evaluation of the lesions.

In this study, integrating diagnostic anatomy into the clinical situation allowed for the determination of the anatomical localization of the lesion (which vertebral segment it is in), improved interpretation of radiographic images, better surgical planning, selection of surgical approaches, selection of safe anatomical corridors, and protection of neurovascular structures. Furthermore, thanks to the diagnostic anatomical approach, the dimensions of anatomical structures were evaluated, and the appropriate implant type and size were determined. Finally, the risk of complications was reduced. Knowledge of anatomical variations and critical structures such as the aorta and caudal vena cava contributed to the prevention of iatrogenic injuries. Additionally, spinal cord compression or implant-related vertebral fractures can be prevented by placing the screws in the correct location.

## Figures and Tables

**Table 1:** Grading of neurological symptoms according to Grasmueck and Steffen (2004) [17].

Grade	Neurological Dysfunction	Number of Cats
I	No back pain or neurological deficits	0
II	Ambulatory paraparesis, normal urination	0
III	Ambulatory paraparesis, urinary retention	3 (15%)
IV	Non-ambulatory paraparesis/paraplegia, urinary retention, and deep pain perception	9 (45%)
V	Paraplegia, urinary retention, absence of deep pain perception	8 (40%)

**Table 2:** A summary of the findings for each case.

No.	Grade	Localization	Treatment Applied	Other Findings	Treatment Results		
					Poor	Functional	Good
1	IV	L6-L7	Polyaxial screw			*	
2	V	T13-L1	Polyaxial screw		*		
3	III	L5-L6	SOP			*	
4	IV	L2-L3	SOP			*	
5	V	L6-L7	SOP		*		
6	IV	T13-L1	SOP			*	
7	IV	L3-L4	SOP Dorsal laminectomy				*
8	III	L1-L2	Polyaxial screw			*	
9	V	T12-T13	Polyaxial screw	Radius ulna fracture	*		
10	IV	L7-S1	Polyaxial screw			*	
11	V	T12-T13	Polyaxial screw	Scapula fracture	*		
12	V	T10-T11	Polyaxial screw, dorsal laminectomy	Coxa fracture	*		
13	V	L7-S1	SOP		*		
14	IV	T11-T12	SOP			*	
15	IV	L4-L5	SOP	Ilium fracture		*	
16	IV	L2-L3	Bilateral polyaxial screw, hemilaminectomy			*	
17	V	T11-T12	Polyaxial screw			*	
18	IV	T12-T13	Polyaxial screw			*	
19	IV	L4-L5	SOP				*
20	V	T9-T10	Polyaxial screw		*		

## Data Availability

The original contributions presented in this study are included in the article. Further inquiries can be directed to the corresponding author(s).
